# Phenotypes and trajectories of suicidality in borderline personality disorder: transitions from ideation to non-suicidal self-injury and suicide attempts

**DOI:** 10.1192/j.eurpsy.2026.10687

**Published:** 2026-07-31

**Authors:** D. Jelaga, M. Belous

**Affiliations:** Department of Mental Health, Medical Psychology and Psychotherapy, Nicolae Testemițanu State University of Medicine and Pharmacy, Chisinau, Moldova, Republic of

## Abstract

**Introduction:**

In adults with borderline personality disorder, lifetime suicidal ideation, non-suicidal self-injury and suicide attempts are frequent. Recent syntheses report approximately 80% ideation, 52% attempts and 6% death by suicide; adult transitions along the ideation to non-suicidal self-injury to attempt pathway remain incompletely quantified.

**Objectives:**

(1) Quantify adult prevalence percentages for ideation, non-suicidal self-injury and attempts; (2) characterise day-level ideation in attempters versus non-attempters; (3) examine associations between diagnostic criteria (including self-harm and chronic emptiness) and attempts.

**Methods:**

Searches were run in MEDLINE/PubMed, Embase, PsycINFO and Web of Science (2020–2025). Inclusion: adults with clinician-diagnosed BPD; observational/longitudinal studies, ambulatory/ecological monitoring, meta-analyses and systematic/narrative syntheses reporting outcomes. Exclusion: samples exclusively <18 years; mixed samples without a dedicated subgroup; no outcomes; duplicates. Flow: full texts assessed, 15; included, 8; excluded, 7 (minors n=3; mixed without subgroup n=2; no outcomes n=1; duplicate n=1). Populations were adult clinical and community samples; age/sex were not consistently reported. Outcomes: ideation, non-suicidal self-injury, attempts, suicide deaths.

**Results:**

Aggregate clinical estimates were approximately 80% lifetime ideation, 52% lifetime attempts and 6% suicide deaths. In a representative adult sample (n=36,309), lifetime attempts among those with a lifetime diagnosis were 22.7% and past-year 3.0%; a sensitivity analysis excluding the diagnostic self-harm criterion yielded 28.1% and 3.0%. Diagnostic self-harm and chronic emptiness remained independently associated with attempts (OR up to 24.28 for self-harm). In 21-day ambulatory monitoring, 66.7% reported at least one day with ideation; day-level ideation occurred on 10.8% of days (6.5% among non-attempters versus 19.5% among attempters). Non-suicidal self-injury was common in adult clinical samples ( range 65–80%) and associated with attempts; in a veteran cohort, non-suicidal self-injury (48%) remained the sole independent correlate of lifetime attempts (adjusted OR 4.9).

**Conclusions:**

Evidence supports a common adult trajectory from ideation to non-suicidal self-injury to attempt, with patterns associated with lethality. The magnitude of percentages and the independent association of non-suicidal self-injury with attempts support time-sensitive risk assessment and targeted interventions (reducing non-suicidal self-injury, enhancing decisional flexibility, managing acute interpersonal stressors) to interrupt progression towards lethal behaviours. A main limitation is that state-to-state transition probabilities were often not reported; between-study heterogeneity was substantial and publication/reporting bias cannot be excluded.

**Disclosure of Interest:**

None Declared

